# Primary Small Lymphocytic Lymphoma of the Renal Capsule: A Histopathological Case Report

**DOI:** 10.7759/cureus.6428

**Published:** 2019-12-20

**Authors:** Hristo Popov, Ina Kobakova, George S Stoyanov

**Affiliations:** 1 General and Clinical Pathology, Forensic Medicine and Deontology, Medical University of Varna, Varna, BGR

**Keywords:** primary renal lymphoma, hematology, small lymphocytic lymphoma, pathology

## Abstract

Lymphomas are one of the most common malignant entries across all populations. Originating most commonly from the lymph nodes, extranodal lymphomas, including those originating from the connective tissue capsule of the internal organs, are rare entries. Herein we present a case report of a 78-year-old male patient presenting with a palpable mass in the region of the left kidney. Ultrasound and computer tomography revealed a mass engulfing the kidney. Nephrectomy was performed, with the histopathological investigation revealing a non-Hodgkin lymphoma originating from the renal capsule, with infiltration into the adjacent tissues. The patient was referred to a hematologist for treatment and one and a half years later, following two negative bone marrow biopsies, the patient is alive and disease-free.

## Introduction

Lymphomas are consistently cited as amongst the top 10 most common malignancies, with increasing incidence worldwide [1,2]. Originating primarily from the lymph nodes, tonsils, and mucosa-associated lymphoid tissue, lymphomas can also seldom originate as primary lesions in parenchymal organs such as the spleen and liver and more rarely in the lung and central nervous system.

Primary lymphoma of the connective tissue capsule of parenchymal organs is an extremely rare finding and can often be interpreted as a sign of infiltration or a soft tissue sarcoma [3].

## Case presentation

**Figure 1:**
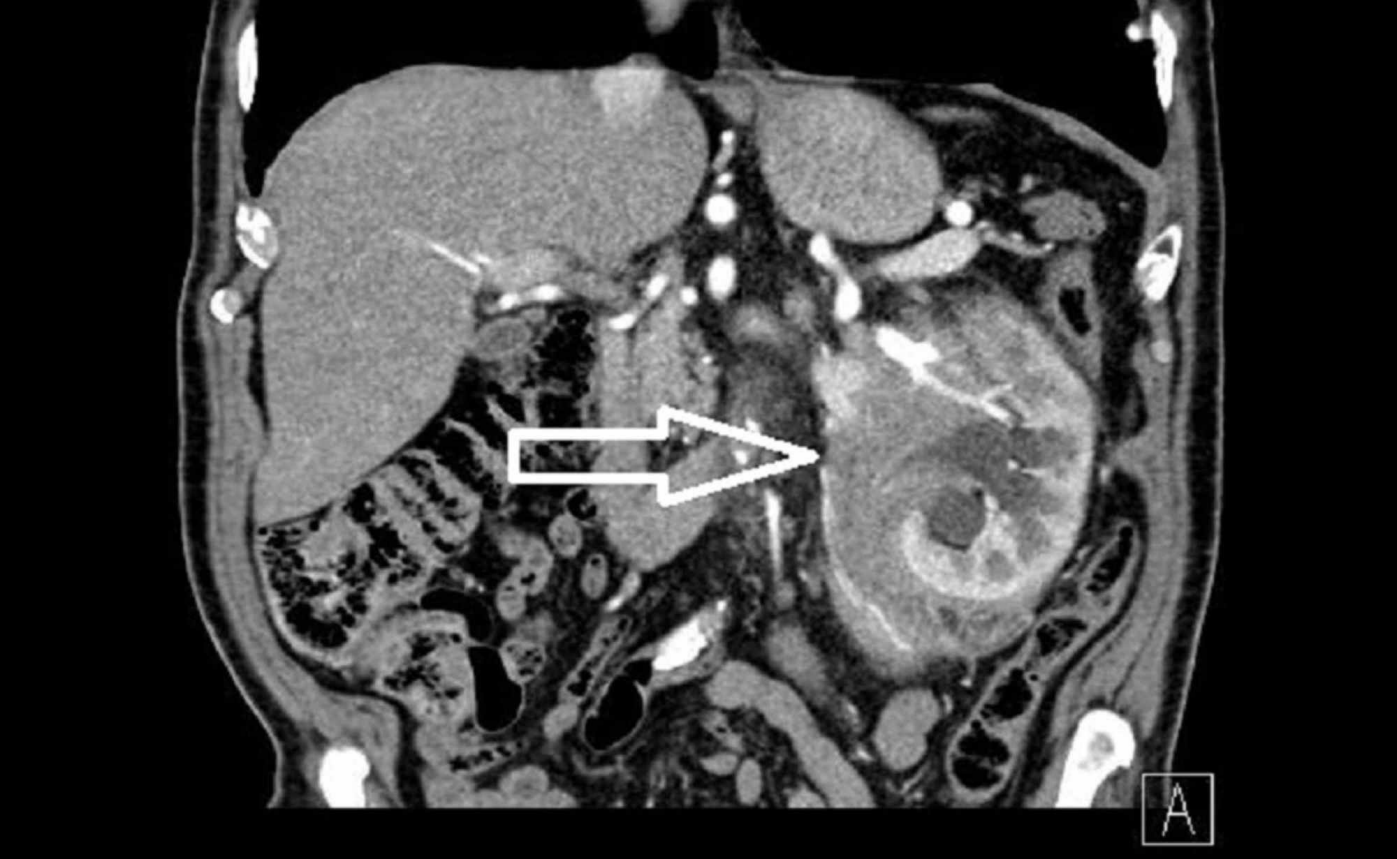
Computed tomography finding (arrow)

Herein we report the findings in a 78-year old male. The patient presented with a palpable mass in the left renal region, without any further complaints such as pain, hematuria, and weight loss. An ultrasound and computer tomography scan showed a large formation in the renal capsule (Figure 1). Concomitant diseases and past medical history included four traumatically fractured ribs, healed without complications, hypertension, angina pectoris, and an aortocoronary bypass.

An endoscopic nephrectomy approach was chosen. The procedure was carried out under general anesthesia. An additional renal artery and vein were located in the inferior pole and after their ligation and separation, the principal renal artery and vein were ligated and separated. An extended resection was performed with full ureter resection and partial resection of the urinary bladder. The kidney along with its capsule was then dissected and explanted through a subcostal incision.

**Figure 2:**
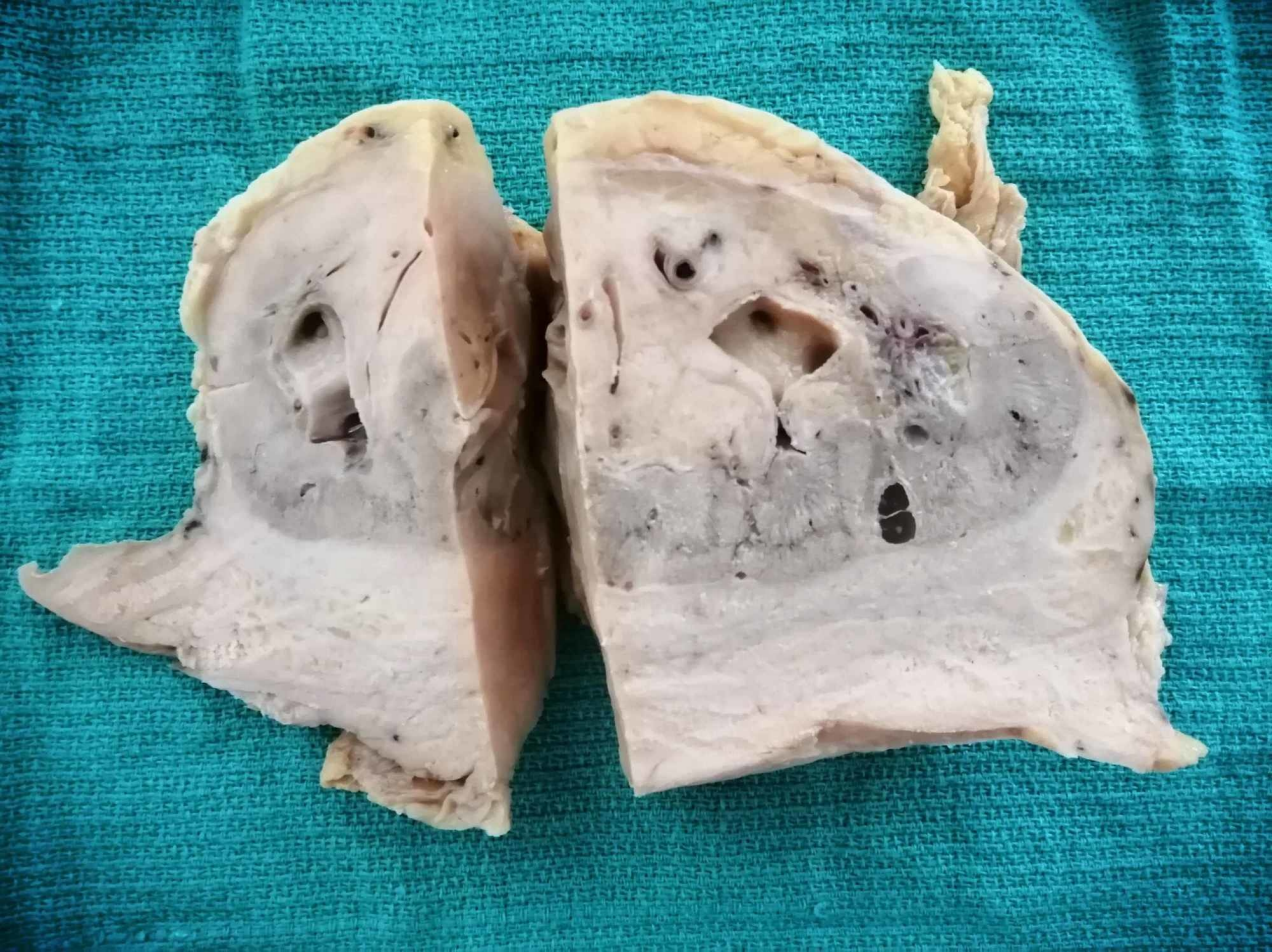
Gross view of the specimen

The materials were sent for histopathological evaluation. The total specimen measured 17/10 cm, of which the kidney specimen measured 12/7 cm and the adjacent pale and solid tumor tissue measured 10/1.5 cm (Figure 2). The adjacent ureter had a length of 15 cm. The specimens were fixed in 10% neutral-buffered formaldehyde and embedded in paraffin (FFPE) for staining with hematoxylin and eosin (H&E) and immunohistochemistry (IHC).

**Figure 3:**
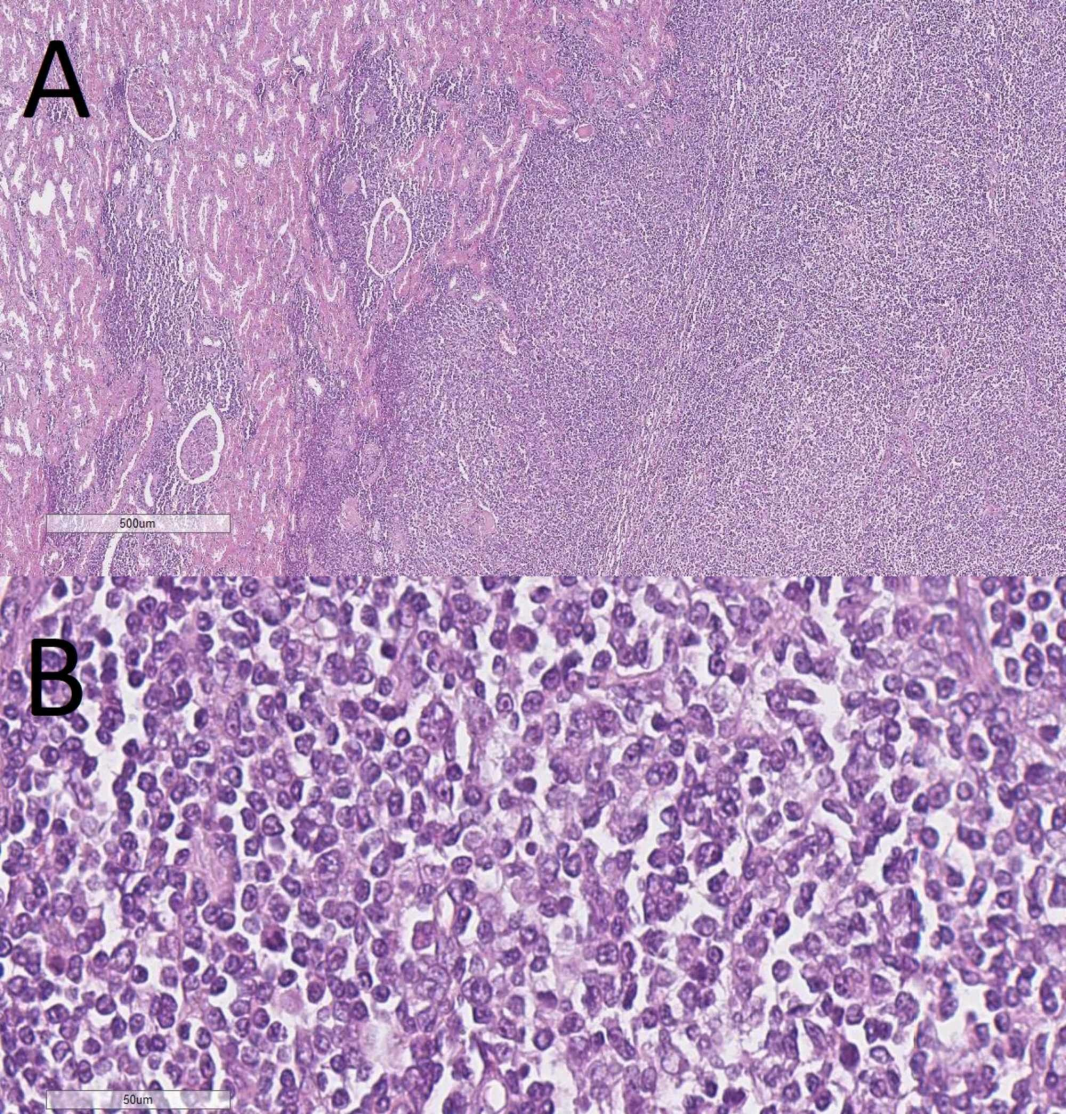
Lymphocytic proliferation in the renal capsule with infiltration into the parenchyma, H&E stain, (A) original magnification 40x, (B) original magnification 400x H&E: hematoxylin and eosin

**Figure 4:**
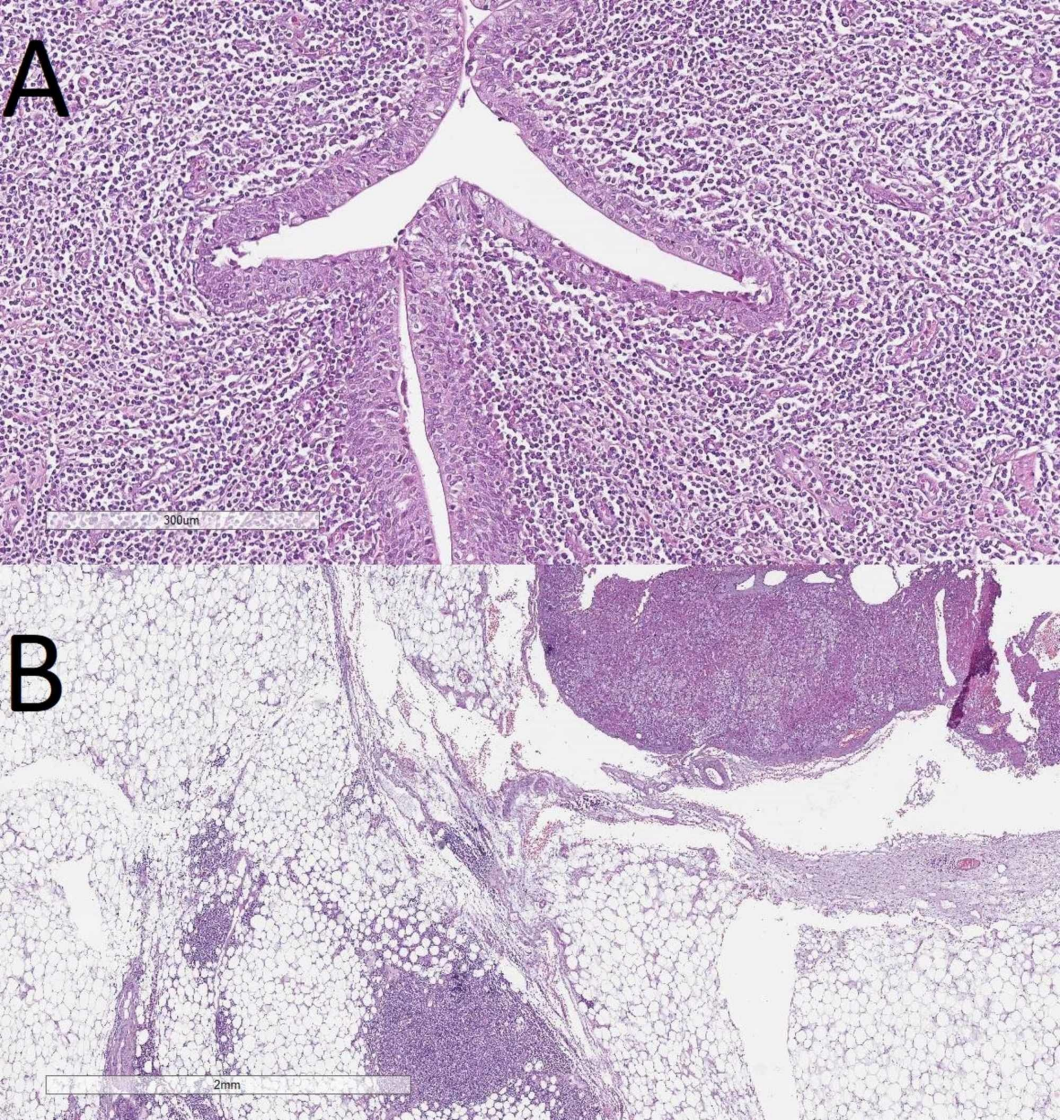
(A) Invasion into the proximal part of the ureter, H&E, original magnification 100x. (B) Invasion into the periadrenal connective tissue, H&E, original magnification 20x H&E: hematoxylin and eosin

**Figure 5:**
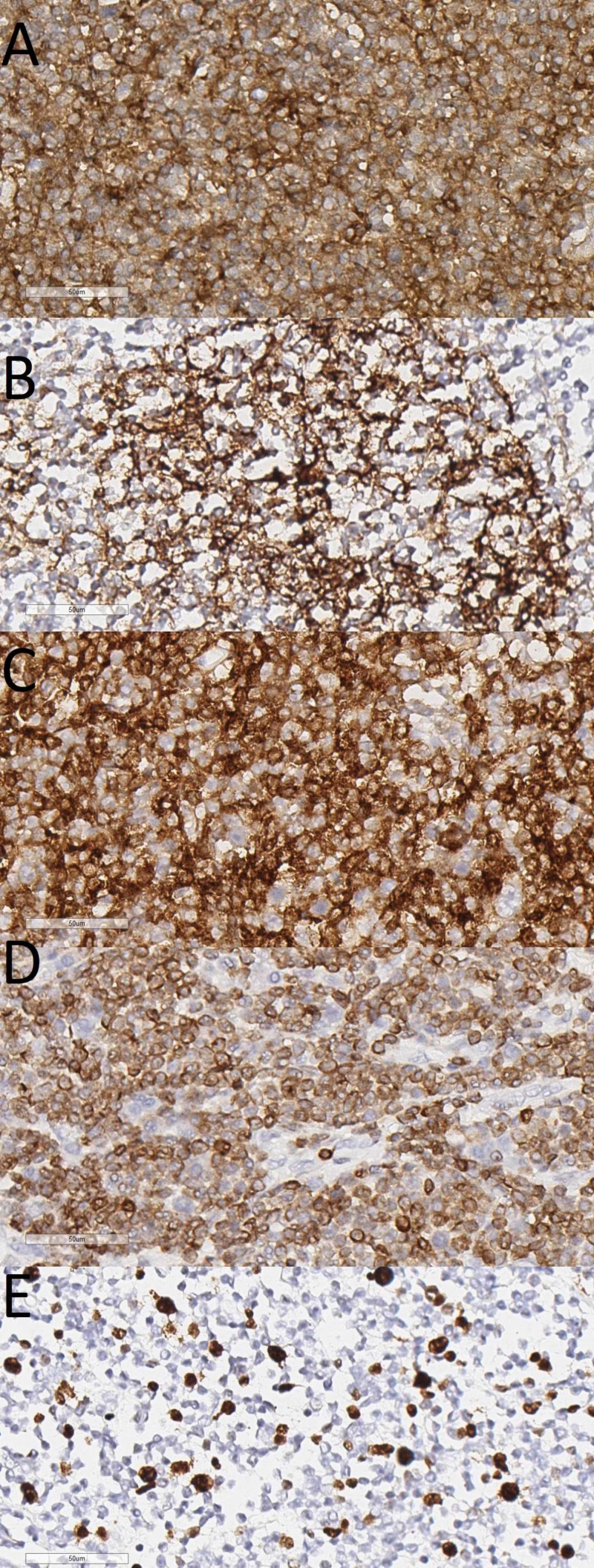
IHC profile (A) CD43, original magnification 400x; (B) CD23, original magnification 400x; (C) CD5, original magnification 400x; (D) BCL2, original magnification 400x; (E) Ki-67, original magnification 400x IHC: immunohistochemistry; CD: cluster of differentiation; BCL2: B-cell lymphoma 2

The FFPE tissue sections stained with H&E showed nodular structures comprised of small to medium-sized atypical monomorphous lymphoid cells with formation of pseudofolicles. The tumor originated from the renal capsule and infiltrated the kidney, adjacent adrenal gland capsule, renal hilar vessels, and proximal part of the ureter, without infiltration into its distal part and the accessory renal vessels (Figures 3, 4). The adjacent perirenal lymph nodes were not involved in the process. The tumor cells were showed a positive cytoplasmic B-cell lymphoma 2 (Bcl2), and a membranous cluster of differentiation 3 (CD3), CD5, CD20, CD23, and CD43 IHC reaction (Figure 5). The tumor cells were negative for Cyclin D1 and Bcl6, whilst CD10 and CD30 reacted only with a small number of scattered cells within the tumor sample. The Ki-67 index for positive nuclei amounted to 30%.

Despite the casuistical origin, based on the lack of other affected structures and both the H&E tumor sample and the reactions with the IHC panel, the diagnosis of small lymphocytic lymphoma was accepted.

The postoperative period was uneventful. The patient was referred to a hematologist for treatment and subsequent reevaluation. Two additional bone marrow biopsies were performed, with no evidence for bone marrow invasion by the process. One and a half years postoperatively, the patient is alive and disease-free.

## Discussion

Hematological malignancies require deep-rooted knowledge for their interpretation and a wide set of IHC markers. In the case of extranodal lymphomas, the interpretation is even more difficult, and the histology needs to be interpreted together with the radiological findings and the patient’s clinical data to differentiate from infiltration from a nodal lymphoma [4].

In such cases, a bone marrow biopsy should also be performed to evaluate the progression of the disease and also rule out leukemia with infiltration into the parenchymal organs.

Differential diagnosis with small round blue cell tumors, more often originating from the kidney and perirenal tissue should also be kept in mind, which further prioritizes the use of IHC [5,6]. These tumors include all types of soft tissue sarcomas, and the undifferentiated types among them, such as liposarcoma and rhabdomyosarcoma, may mimic the histological profile of lymphoma. In male patients, they also mimic germ cell tumors that originate from an undescended testis.

The value of IHC in such cases is unquestioned as, despite the similarities in the histological profile, the IHC reactions are specific for the separate groups of tumors such as CD20 for B-cell lymphomas, OCT3/4 in germ cell tumors, and S100 in soft tissue sarcomas.

The kidneys, however, are the most common site of extranodal lymphomas, although most commonly affected as a secondary site [7]. As such special emphasis should always be placed on renal lymphoma, and especially a multidisciplinary approach should be taken in such patients to differentiate between a primary entry and secondary involvement [7].

## Conclusions

Primary lymphoma of the renal capsule is a rare condition, requiring a wide set of IHC markers and interpretation together with the radiological finding, clinical data, and bone marrow biopsy to differentiate it from nodal lymphoma infiltration and small round blue cell neoplasms.
